# Ventricular Endocardial Tissue Geometry Affects Stimulus Threshold and Effective Refractory Period

**DOI:** 10.1016/j.bpj.2018.11.003

**Published:** 2018-11-09

**Authors:** Adam Connolly, Allen Kelly, Fernando O. Campos, Rachel Myles, Godfrey Smith, Martin J. Bishop

**Affiliations:** 1 Department of Bioengineering, School of Biomedical Engineering and Imaging Sciences, King’s College London, London, United Kingdom; 2 Institute of Cardiovascular and Medical Sciences, University of Glasgow, Glasgow, United Kingdom

## Abstract

Background: Understanding the biophysical processes by which electrical stimuli applied to cardiac tissue may result in local activation is important in both the experimental and clinical electrophysiology laboratory environments, as well as for gaining a more in-depth knowledge of the mechanisms of focal-trigger-induced arrhythmias. Previous computational models have predicted that local myocardial tissue architecture alone may significantly modulate tissue excitability, affecting both the local stimulus current required to excite the tissue and the local effective refractory period (ERP). In this work, we present experimental validation of this structural modulation of local tissue excitability on the endocardial tissue surface, use computational models to provide mechanistic understanding of this phenomena in relation to localized changes in electrotonic loading, and demonstrate its implications for the capture of afterdepolarizations.

Methods and Results: Experiments on rabbit ventricular wedge preparations showed that endocardial ridges (surfaces of negative mean curvature) had a stimulus capture threshold that was 0.21 ± 0.03 V less than endocardial grooves (surfaces of positive mean curvature) for pairwise comparison (24% reduction, corresponding to 56.2 ± 6.4% of the energy). When stimulated at the minimal stimulus strength for capture, ridge locations showed a shorter ERP than grooves (*n* = 6, mean pairwise difference 7.4 ± 4.2 ms). When each site was stimulated with identical-strength stimuli, the difference in ERP was further increased (mean pairwise difference 15.8 ± 5.3 ms). Computational bidomain models of highly idealized cylindrical endocardial structures qualitatively agreed with these findings, showing that such changes in excitability are driven by structural modulation in electrotonic loading, quantifying this relationship as a function of surface curvature. Simulations further showed that capture of delayed afterdepolarizations was more likely in trabecular ridges than grooves, driven by this difference in loading.

Conclusions: We have demonstrated experimentally and explained mechanistically in computer simulations that the ability to capture tissue on the endocardial surface depends upon the local tissue architecture. These findings have important implications for deepening our understanding of excitability differences related to anatomical structure during stimulus application that may have important applications in the translation of novel experimental optogenetics pacing strategies. The uncovered preferential vulnerability to capture of afterdepolarizations of endocardial ridges, compared to grooves, provides important insight for understanding the mechanisms of focal-trigger-induced arrhythmias.

## Introduction

Initiation of electrical propagation in the heart requires a localized region of depolarizing current (source) of sufficient magnitude to overcome current drain (sink) from the surrounding, electrically coupled tissue (1, 2). This interplay between source and sink is critical during onset of the heartbeat, as well as initiation (and termination) of pathological arrhythmic activity. During sinus rhythm, small areas of tissue, such as the sinoatrial node or Purkinje fibers, must excite the surrounding myocardium to transmit electrical activation throughout the heart in a coordinated manner. Under pathological conditions, isolated groups of myocytes may spontaneously depolarize (through early or delayed afterdepolarizations (3)), causing triggered activity that may excite neighboring tissue, inducing an arrhythmia. Despite their acknowledged presence and importance at the single-cell level, how isolated afterdepolarization events successfully capture neighboring tissue and overcome the significant electrotonic current drain present in normal well-coupled myocardium still remains largely unresolved (2).

As well as physiological or pathological spontaneous tissue capture, controlled external electrical stimulation from surface fine-wire electrodes is used to pace myocardial tissue during preclinical experiments on both in vivo and ex vivo animal preparations, allowing a variety of protocols to be administered for mechanistic investigation of different electrophysiological phenomena. There is also an increasing interest in the potential of biological pacemakers from the field of optogenetics, which may allow controlled regions of myocardium to be stimulated using directed surface light sources (4, 5, 6). Developing a comprehensive biophysical understanding of local surface stimulation of cardiac tissue and its dependence upon local tissue anatomy is essential to help further develop such novel pacing strategies currently in the experimental phase.

Whether a localized pacing stimulus successfully captures the neighboring myocardium depends upon the amount of stimulus current delivered (its strength and duration (7)) and the state of excitability of the tissue at the site of delivery, as well as the electrotonic loading imposed on the stimulated region by the surrounding electrically coupled tissue. Electrotonic current is the term given to the diffusive flow of charge from myocyte to myocyte (through gap junctions) responsible for mediating the spatial spread of the electrical excitation wave throughout cardiac tissue. The electrotonic loading imposed on a particular region (the “source”) represents the magnitude of the downstream current required to be passed on to downstream tissue (the “sink”) to successfully continue or initiate the spread of electrical propagation. Electrotonic loading may be modulated by wavefront curvature (8), alterations in local electrical coupling (2, 9, 10), or changes in anisotropy (10) and is also significantly influenced by effective changes in tissue dimensionality, for example, at the junction of a Purkinje fiber and coupled myocardium (11) and at the mouth of a rapid funnel-like tissue expansion (12, 13).

Recent modeling studies by our group (12, 14) have shown how intramural tissue boundaries, because of microanatomical structures, alter electrotonic loading and can significantly influence the capture of focal stimuli occurring within the myocardium. Simulations in tissue with homogeneous ionic properties showed that effective refractive periods (ERPs) for focal stimulus capture applied in the neighborhood of blood vessel cavities were up to 40 ms lower compared to remote well-coupled tissue, despite having almost identical action potential durations (APDs) (14). We have also demonstrated in a combined experimental and modeling study that progressively diminished electrotonic loading as a wavefront approaches a sealed tissue boundary (the epicardium) (15) progressively reduces the downstream axial current, decreasing action potential upstroke duration. The presence of fibrosis may provide similar sealed boundaries to current flux. Simulations have shown that this gives rise to lower electrotonic loading, significantly reducing ERP at the mouth of funnel-like tissue expansions within the border zone of infarcted regions (12). The subsequent existence of sharp ERP gradients in both studies were shown to facilitate unidirectional conduction upon appropriately timed focal stimuli, highlighting the importance of microanatomical structure in reentry initiation driven by electrotonic loading. Despite our previous modeling studies, direct experimental observation of the effects on tissue excitability of structurally driven changes in electrotonic loading has not yet been achieved.

In this study, we conduct a combined experimental and computational investigation into the differences in stimulus capture and ERP applied to geometrically distinct spatial locations on the endocardial surface—specifically, endocardial grooves and ridges. Such stimulus locations were chosen because the presence of positive/negative surface curvature in these regions provides differing environments (16, 17) for the boundary confinement of the stimulus current. The endocardium is also accessible via experimental electrodes placed on the tissue surface itself and represents an important location for placement of catheters in the electrophysiology catheter laboratory and in experimental animal studies. It is shown computationally and experimentally that the threshold stimulus strength for excitation propagation depends on the geometry of the surface undergoing stimulation. In the experimental rabbit wedge preparations, the difference in stimulation threshold is small but significant; corresponding similar differences in ERP are also shown. Idealized numerical results agree qualitatively with the experimental results, providing confidence that the mechanism behind the experimental observations is indeed due to boundary confinement. Application of simulations of delayed afterdepolarization (DAD) formation within the same computational models goes on to show how such differences in electrotonic loading induced by the differing boundary structures on the endocardium can alter the preferential location of focal ectopic beats. Our results provide important knowledge regarding the biophysical nature of successful stimulus capture in physiological, pathological, and interventional scenarios.

## Methods

### Theory of boundary confinement

**Figure 1:**
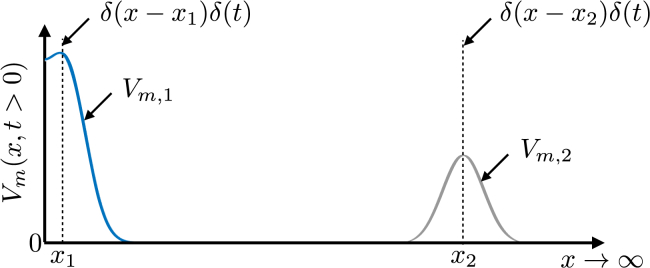
Impulse response of a semi-infinite strand of passive cardiac tissue. The tissue is stimulated with the same strength impulse at two separate locations, *x*_1_, *x*_2_. Location *x*_1_ is close to a sealed boundary (*x* = 0) to current flux, thus having a reduced electrotonic loading on one side; *x*_2_ is far away from any boundary $\left(x_{2}\gg x_{1}\right)$, being well coupled on either side with high electrotonic loading. Note that this plot shows separate solutions for the impulse response applied to the two sites; they are plotted on the same graph for convenience of comparison. To see this figure in color, go online.

In the case of focal stimuli, high electrotonic loading strongly drains current away from the site of stimulation, meaning less current is available to bring the stimulated region to threshold and successfully activate it. Thus, for a given stimulus current density, a region of higher loading requires the tissue to be fully excitable; on the contrary, regions of reduced loading confine the diffusive current and may be excited by a focal stimulus when initially in a more refractory state (at earlier coupling intervals). To illustrate this effect, consider the impulse response of a semi-infinite strand of passive cardiac tissue, as shown in Fig. 1. Stimulation at *x*_1_ near the sealed boundary (at *x* = 0) produces a higher impulse response (higher local *V*_*m*_) compared to stimulation (using the same strength impulse) at *x*_2_, far away from any boundary. This effect may be considered as being due to the reduced electrotonic loading imposed on site *x*_1_ because of the presence of the boundary, leading to confinement of the stimulus current.

In this work, we achieve the boundary confinement effect by stimulating the surfaces of curved regions of tissue: negative curvature boundaries lead to greater boundary confinement than positively curved regions, as first described in (16), and impulse stimuli were approximated by 2 ms duration extracellular potential clamps applied proximal to one another on the tissue surface (discussed further below).

### Computational methods

The finite element solver CARP (Cardiac Arrhythmia Research Package) (18) was used to solve the bidomain partial differential equations for cardiac electrodynamics (19). The bidomain equations, stated in parabolic form, are written as

(1) $$\begin{array}{c} \nabla \cdot \left(\sigma_{i}\nabla \phi_{i}\right)=\beta I_{m},\\ \nabla \cdot \left(\sigma_{e}\nabla \phi_{e}\right)=-\beta I_{m},\\ I_{m}=C_{m}\frac{\partial V_{m}}{\partial t}+I_{ion}\left(V_{m},\eta \right),\\ \sigma_{b}\nabla^{2}\phi_{b}=0, \end{array}$$

where *ϕ*_*i*_ and *ϕ*_*e*_ are the intra- and extracellular potentials, *ϕ*_*b*_ is the bath potential, *V*_*m*_ = *ϕ*_*i*_ − *ϕ*_*e*_ is the transmembrane potential, ***σ***_*i*_ and ***σ***_*e*_ are the intra- and extracellular conductivity tensors, *σ*_*b*_ is the bath conductivity, *β* = 0.14 *μ*m^−1^ is the membrane surface area/volume ratio, *I*_*m*_ is the transmembrane current density, *C*_*m*_ = 1 *μ*F/cm^2^ is the membrane capacitance per unit area, and *I*_*ion*_ is the membrane ionic current density as a function of the transmembrane potential *V*_*m*_ and the vector of state variables ***η***. The boundary conditions imposed on Eq. 1 are

(2) $$\begin{array}{c} n\cdot \left(\sigma_{i}\nabla \phi_{i}\right)=0,\;\partial \Omega_{t}\\ n\cdot \left(\sigma_{e}\nabla \phi_{e}\right)=\sigma_{b}n\cdot \nabla \phi_{b},\;\partial \Omega_{tb},\\ \sigma_{b}n\cdot \nabla \phi_{b}=0,\;\partial \Omega_{b}, \end{array}$$

where ***n*** is the outward pointing unit-normal to the specific boundary and *∂Ω*_*t*/*b*/*tb*_ is the boundary of the tissue/bath/tissue-bath interface. In the simulations, we did not include a bath space surrounding the myocardium (meaning the last equation in Eq. 2 was not explicitly solved); instead, dissimilar Dirichlet terms were applied to the extracellular space coincident with the tissue surface.

The intra and extracellular conductivities were assigned experimentally measured values (20) of $g_{i}^{l}=0.17$, $g_{i}^{t}=0.019$, $g_{e}^{l}=0.62$, and $g_{e}^{t}=0.24$, giving transverse and longitudinal space constants (21) of *λ*_*t*_ ≈ 355 *μ*m and *λ*_*l*_ = 976 *μ*m, respectively. The ionic currents *I*_*ion*_ were described by a rabbit ventricular cell model (22) and were integrated with the Rush-Larsen scheme (23) using a global time step of 20 *μ*s—a value known to give stable and accurate results (24).

### Computational models

**Figure 2:**
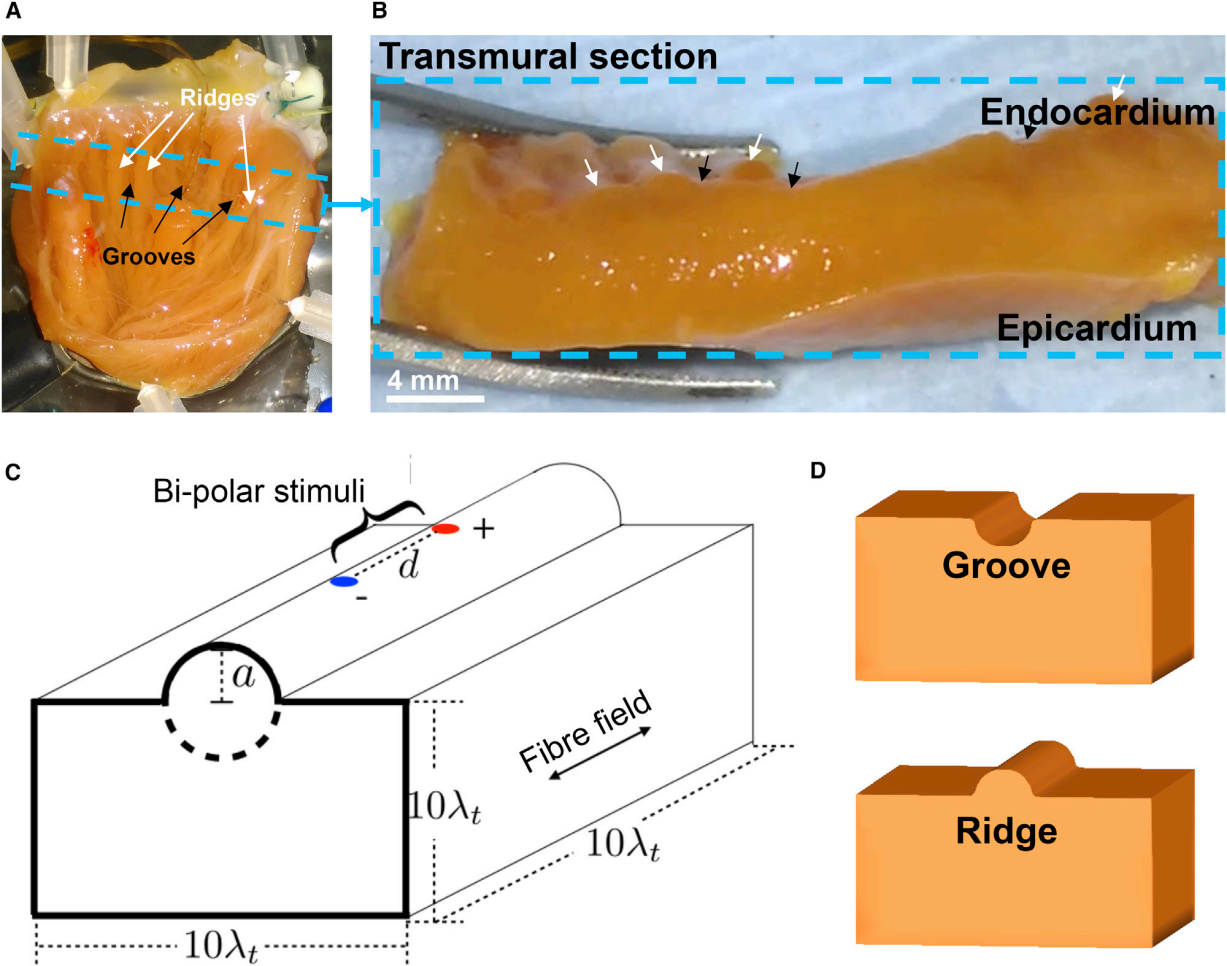
(*A*) Rabbit left-ventricular endocardial surface showing trabeculae. (*B*) A transmural section of the rabbit LV (from the region indicated by the *dashed blue box* in *A*) showing endocardial ridges (*white arrows*) and grooves (*black arrows*) and a smooth epicardial surface. (*C*) A schematic of the computational geometries used. A half-cylinder of radius *a* was either added (*solid line*) or subtracted (*dashed line*) to form a ridge or a groove in the cuboidal domain of side length 10*λ*_*t*_, sufficiently large to neglect boundary effects far from the region of interest. The bipolar stimuli were applied to patches around the midpoint of the ridge/groove and separated by a distance *d*. (*D*) Examples of the resultant computational geometries created—in this case, *a* = 2*λ*_*t*_. To see this figure in color, go online.

A set of idealized three-dimensional (3D) endocardial geometries were considered, consisting of half-cylinders of radius *a* added or subtracted from the top surface of a cuboid of edge-length 10*λ*_*t*_—sufficiently large to neglect boundary effects far from the region of interest. Adding the half-cylinder represents an idealized endocardial ridge, whereas subtracting the half-cylinder represents an idealized endocardial groove, as shown in Fig. 2 *C*. The magnitude of the principle curvature at all points on the cylinder is 1/*a*, and the sign of the principle curvature is negative for the ridges and positive for the grooves. In addition, one further geometry was considered with a flat upper surface (or zero curvature in the limit as |*a*| → *∞*). The domains were discretized with linear tetrahedral finite elements, which were highly refined around the upper surface, giving an element edge length of approximately 5 *μ*m in this region. Cardiac fiber orientation was prescribed to be parallel with the axis of the cylinder, reflecting the fact that fibers are naturally aligned with the axis of trabeculations (25). Examples of the computational geometries created are shown in Fig. 2 *D*.

#### Bipolar stimulus representation

Stimulation of cardiac tissue via a bipolar electrode arrangement (in which anode and cathode are in close proximity (a few *mm*) has been used for many decades. During externally applied bipolar stimulation, current is passed between two poles of an electrode. As current passes between the two poles, it enters and exits through the proximal myocardial tissue, giving rise to local hyper- and depolarization, respectively. This effect causes tissue excitation, which spreads, activating neighboring myocardium. Bipolar electrode stimulation was represented within the model by two small patches (one anodal, one cathodal) of 25 *μ*m radius around the midpoint of the ridge/groove (see Fig. 2 *C*). The patches were separated by a distance *d* = *λ*_*t*_, approximating the separation of the fine bipolar stimulus electrode used experimentally. The stimuli were applied for 2 ms and computationally represented as extracellular potential clamps of ±(*ΔV*)/2 between the anode/cathode, where *ΔV* represents the magnitude of the applied voltage stimulus.

### Experimental methods

All of the experimental methods described here conformed with the guide for the care and use of laboratory animals and were approved by the institutional animal use and care committee.

### Isolated ventricular wedge preparation

Electrophysiology of the left ventricle (LV) endocardial surface was examined using an isolated LV wedge preparation, described in detail previously (26). Eight adult male New Zealand white rabbits (body weight 3–3.5 kg) were sacrificed with an intravenous injection of sodium pentobarbital (1.0 mL) and heparin (0.5 mL, 5000 IU/mL). Hearts were rapidly excised, placed in cold (4°C) Tyrode’s solution, and transferred to a modified Langendorff perfusion system. The heart was suspended vertically on an adjustable metal holder via the remaining portion of the aorta. The aorta was then bisected to allow access to the coronary ostia, and a custom 2F catheter was inserted into the left descending coronary artery before being sutured in place to perfuse the LV with oxygenated 37°C Tyrode’s solution: 116.0 mM NaCl, 20.0 mM NaHCO_3_, 1.0 mM Na_2_HPO_4_, 1.0 mM MgSO_4_, 5.0 mM KCl, 1.5 mM CaCl_2_, 11.0 mM glucose (pH 7.4), maintained by continuous bubbling with 95% O_2_/5% CO_2_ gas mixture. The heart was then dissected until only the LV free wall remained. Tissue was then opened out and pinned horizontally to a silicon cradle. Larger coronary vessels exposed by dissection were tied off to prevent leaks and maintain coronary perfusion pressure between 60 and 80 mmHg. After a 15 min stabilization period, preparations were perfused continuously with 10 *μ*M blebbistatin (Abcam, Cambridge, UK) to inhibit contraction. The endocardial surface of the rabbit LV was sufficiently complex, with endocardial undulations of varying size and spatial separation; however, this pattern was relatively uniform between hearts. To ensure pacing sites were spatially well defined, structures closer to the basal region of the heart were chosen (region indicated by *blue dashed box* in Fig. 2 *A*). Electrodes were placed on ridge structures that were at least 1.5 mm wide and did not contain significant branching within 2 mm either side of the electrode placement. Grooves were chosen as relatively flat regions adjacent to a ridge. Endocardial structures were electrically stimulated using bipolar copper-plated silver wire electrodes (wire diameter, 0.4 mm; interelectrode distance ≈ 1 mm) (lacquer coated for insulation). The uninsulated ends were placed on the endocardial tissue, and the body of the wires (∼20 cm length) was supported by a clamp. The electrode pair was placed parallel to the groove and ridge structure, and care was taken to ensure that there was no mechanical distortion of the tissue surface. A pseudo-electrocardiogram (ECG) was recorded via Ag/AgCl electrodes embedded into the silicon cradle to monitor stimulus capture.

### Optical voltage mapping and electrical stimulation protocols

Action potentials were recorded using an optical mapping setup. Preparations were slowly bolus loaded with 50 *μ*L (2 mM) of the voltage-sensitive fluorophore RH237 (Thermo Fisher Scientific, Waltham, MA) over a 10 min period, introduced via an in-line injection port on the perfusion system. RH237 was excited by four spotlight light-emitting diodes surrounding the preparation (peak excitation wavelength 520 nm). Emitted light was collected by a macro-objective lens (0.63 or 1.0× magnification; Leica, Wetzlar, Germany), split with a dichroic mirror at 660 nm, long-pass filtered at 715 nm, and projected onto a MiCAM Ultima 100 × 100 pixel CMOS camera (Scimedia, Costa Mesa, CA) attached to a 2.0× condensing lens, giving an effective field of view of 31 (0.63×) or 20 (1.0×) mm^2^. All optical signals were sampled at 1 kHz. Preparations were initially stimulated at a basic cycle length of 350 ms (2 ms pulse width). Stimulus electrodes were positioned first on endocardial grooves, then endocardial ridges, with electrodes running parallel to the orientation of endocardial ridges throughout. After electrode placement, stimulus voltage was gradually increased using a DS2A isolated voltage stimulator with a resolution of 0.01 V (Digitimer, Hertfordshire, UK) until the tissue demonstrated entrainment. This was considered the tissue voltage threshold. ERP was determined using a standard S1-S2 pacing protocol, with a steady-state S1 cycle length of 350 ms, in which the S2 stimulus was lowered at 30 s intervals until the preparation no longer reached activation threshold (1 ms temporal resolution).

### Data acquisition and analysis

ECG, coronary perfusion pressure, stimulus trigger, and light-emitting diode gating signals were recorded and digitized continuously by an A/D board (Powerlab 8/30 and LabChart 8.1; ADInstruments, Dunedin, New Zealand) attached to a PC. The ECG and stimulus trigger were also recorded by the MiCAM05 controller during capture of optical signals. Optical mapping signals were analyzed using custom-written software (OPTIQ; Dr. Francis Burton, University of Glasgow, Glasgow, UK). All data are expressed as mean ± standard error. Experimental parameters were compared using paired Student’s *t*-test, with *p* < 0.05 considered significant.

### Stimulation protocol

During the first four experiments, threshold and ERP evaluation was performed randomly in either ridge (*n* = 2) or groove (*n* = 2). After it was determined that this did not influence the outcome for threshold capture, pacing protocols for the remaining four experiments were performed initially on the groove and then on the ridge. This enabled ERP to be measured for ridges both at its own threshold voltage, then at the threshold voltage matching the groove (higher threshold).

#### Capture threshold

In both experiments and simulations, the minimal bipolar voltage applied to the tissue to elicit successful capture of the surrounding myocardium was obtained. Experimentally, the stimulus voltage was gradually increased until the tissue showed entrainment at a pacing cycle length of 1 s, with successful capture being confirmed by the appearance of a subsequent (pseudo-) QRS complex on the ECG. Computationally, the threshold stimulus (the minimal stimulus strength required to elicit wavefront propagation) was calculated using a bisection algorithm (27), with a tolerance of less than 0.01 V.

#### ERP evaluation

Experimentally, ERP was determined using standard S1-S2 pacing until the tissue no longer captured (1 ms temporal resolution), with a steady-state S1 cycle length of 350 ms. Successful capture was assessed by visual inspection of the ECG. Stimulus thresholds corresponding to the previously found (see above) minimal threshold for capture for each specimen at each ridge/groove location were used to calculate the ERP. In addition, four ERPs were calculated in which the stimulus strength was kept constant between the ridge and groove sites.

Computationally, a bisection algorithm was used to compute the ERP as discussed in other works (12, 14), with a tolerance of <1 ms. The stimulus strength was kept constant for all ridge/groove geometries and given a value of 1.01× the maximal capture threshold of all the ridge/groove geometries.

#### Strength-interval curve computation

Strength-interval curves were computed using a similar approach to the capture threshold and ERP analysis, above (27). Specifically, a single paced beat (S1) was initiated in each computational model, using a stimulus strength equal to the previously computed capture threshold. The state of the tissue was subsequently saved at 10 ms intervals during the refractory period. Simulations were restarted at each saved state, and an S2 stimulus was immediately delivered. A bisection algorithm was again used to determine the strength of stimulus required to elicit tissue capture at this specific state of refractoriness.

### Computational modeling of DADs

The computational modeling approach described above was further augmented to provide a detailed representation of spontaneous calcium release (SCR)-induced DADs. Briefly, an additional stochastic phenomenological mathematical model of SCR events (28) was coupled to the rabbit ventricular cell model (22). In this phenomenological model, an SCR event is represented as a calcium wave that is nucleated in the cell and then propagates in a fire-diffuse-fire way (28). The phenomenological model accounts for experimentally observed features of SCR events and has a dependence on subcellular calcium concentration, ensuring that SCR events are more likely to occur as the cell’s sarcoplasmic reticulum becomes overloaded (29). Following our previous computational studies (30, 31, 32), key parameters of the rabbit ventricular cell model were modified to increase its propensity for calcium-mediated DADs. Specifically, calcium overload was induced by increasing extracellular calcium concentration from 1.8 to 4 mmol/L, average conductance of ryanodine clusters was increased by a factor of 4, the strength of the electrogenic sodium-calcium exchange current was doubled, and the maximal conductance of the inward rectifier potassium current was decreased to 30% of its control value. It is noted that with these ionic changes imposed in the cell model, DAD capture rate is 100% at the single-cell level, as reported in our previous work (31).

To focus the analysis of DAD occurrence on the specific region of interest and to avoid DAD capture of nonphysiological tissue regions in the model (edges/corners, for example, where electrotonic loading is artificially lowered), only tissue within the center of the geometry was made DAD-prone, including a layer of 100 *μ*m beneath the tissue surface. This ensured a consistent volume of DAD-prone tissue was presented in each model to facilitate direct comparison. Simulations to test for DAD capture were performed in line with previous studies (30, 31, 32). Capture of DADs in 3D well-coupled myocardium is known to be highly unlikely (2) because of the significant source-sink mismatch (2), with successful capture of these events requiring significant synchronization of Ca overload. To see stochastic DAD events within our 3D models, we significantly reduced tissue conduction (to ∼7%), replicating highly severe pathological heart-failure-like conditions in which DAD capture may be most likely. This was done uniformly in all groove and protrusion models, thus allowing us to directly compare the effects of differences in electrotonic loading on capture of DADs. Briefly, in each model, 100 simulations were performed in which the tissue was initially paced in space-clamped mode at a cycle length of 500 ms. Simulations were then restarted at the tissue level, 500 ms after the last paced beat, followed by 1000 ms of diastole. Should capture of the tissue by a DAD event occur within this time, its location was noted and the simulation terminated. This allowed a statistical representation of the likelihood of DAD capture to be built-up for each geometry considered.

## Results

### Capture threshold

**Figure 3:**
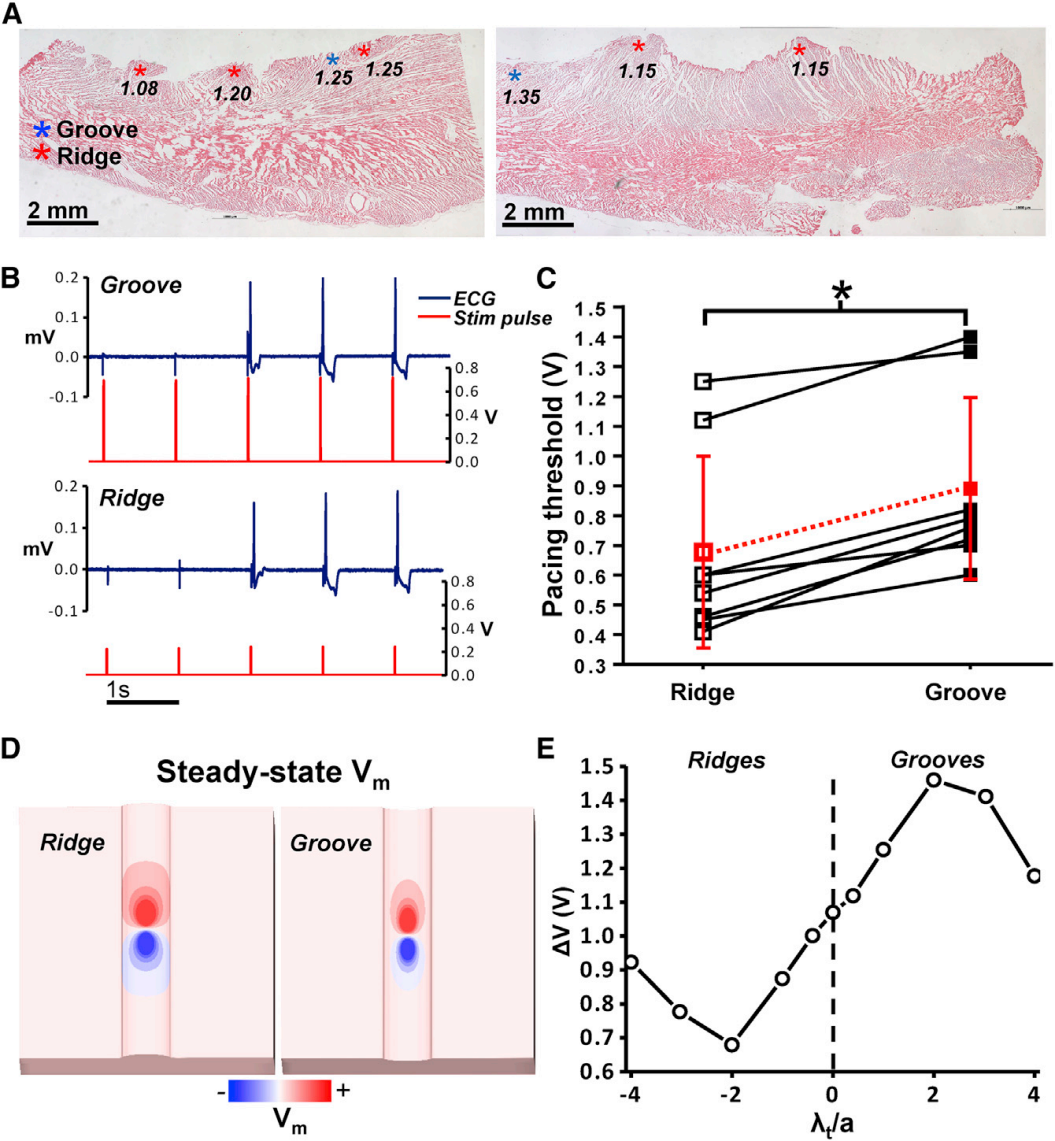
(*A*) Histological slices showing locations of experimental measurements of stimulus thresholds. Note that the histological processing distorted the (previously prominent) endocardial geometry. (*B*) Representative ECG (*blue trace*) and stimulus pulse (*red trace*) records from a perfused LV wedge preparation. The bipolar stimulus strength was gradually increased at a pacing cycle length of 1 s until tissue capture was achieved, as shown by the large deviation (pseudo-QRS complex) in the ECG trace. (*C*) The pacing thresholds at ridge and groove stimulus sites in each experimental preparation; the mean pacing threshold is shown by the red dotted line. The ^∗^ indicates a statistically significant result (*p* < 0.001). (*D*) The computational steady-state transmembrane potential (with a passive membrane) for the |*λ*_*t*_ /*a*| = 2 ridge and groove; the spatial decay in the virtual-electrode magnitude is more rapid for the groove because of the less pronounced effect of boundary confinement. (*E*) The computational bipolar stimulus threshold (in volts) for ridge and groove geometries as parameterized by the nondimensional curvature *λ*_*t*_/*a*. Note that the dotted line at zero curvature represents a flat surface. To see this figure in color, go online.

Fig. 3 *A* shows histological analysis of one LV wedge preparation, highlighting the locations at which stimulation was performed along with the corresponding voltage thresholds for capture. These images highlight the diversity of endocardial trabecular structures and were used to inform computational model generation.

#### Experimental results

Fig. 3 *B* shows the ECG traces during progressive testing for stimulus capture in the groove (*upper trace*) and ridge (*lower trace*). Fig. 3 *C* then shows the actual values of capture threshold for each experiment (*black squares*), with mean data also shown (*red squares*). The mean minimal stimulus strength required to elicit successful capture for all preparations for the ridge (0.68 V) is lower than that of the groove (0.89 V), approximately a reduction of 24%, equivalent to 57.8% of the energy (proportional to *V*^2^). However, the variation in baseline capture threshold between individual preparations was high, as shown by the relatively large error bars in Fig. 3 *C*. Therefore, comparing ridge versus groove capture thresholds for individual preparations (in a pairwise manner) shows that the minimal stimulus strength required to elicit successful capture from an endocardial ridge is consistently and significantly lower than that required to capture from a corresponding groove for all preparations. The pairwise mean difference in capture threshold was 0.21 ± 0.03 V (*p* < 0.001), comparing groove threshold minus ridge threshold.

#### Computational results

Computational comparison of this reduction in stimulus threshold within a ridge compared to a groove is shown in Fig. 3, *D* and *E*. Fig. 3 *D* shows the steady-state transmembrane potential field on the surfaces of a ridge and groove for |*λ*_*t*_/*a*| = 2. Proximal regions of depolarization (under the cathode) and hyperpolarization (under the anode) are clearly visible in both cases. In the case of the groove, the elevated voltage under the cathodal stimulus site decays at a faster rate along the axis of the structure as opposed to the ridge. This effect is caused by the increased confinement of the stimulus current in the case of the ridge, relative to the groove.

Fig. 3 *E* shows the nondimensional curvature of the geometry (*λ*_*t*_/*a*) versus the threshold stimulus strength (*ΔV*) required to elicit wavefront propagation from the bipolar stimulus applied to each of the geometries considered. The shape of the curve implies that negative curvature boundaries (representing protrusions) require a lower threshold stimulus than positive curvature boundaries (representing grooves). As the curvature of the cylinder becomes more negative, the threshold stimulus decreases and then increases for low negative curvatures. As the curvature becomes positive, the threshold stimulus increases and then decreases for high positive curvatures. In other words, as the magnitude of the curvature |*λ*_*t*_/*a*| → ∞, the threshold stimulus approaches the zero-curvature value (i.e., the value at *λ*_*t*_/*a* = 0); this is because as *a* → 0, although the curvature increases, the size of the cylinder decreases and the electrotonic effect of the cylindrical boundary reduces commensurately. The extrema in the threshold stimuli were around |*λ*_*t*_/*a*| ≈ 2, corresponding to a cylinder diameter of approximately *λ*_*t*_.

### Effective refractory period

#### Experimental results

**Figure 4:**
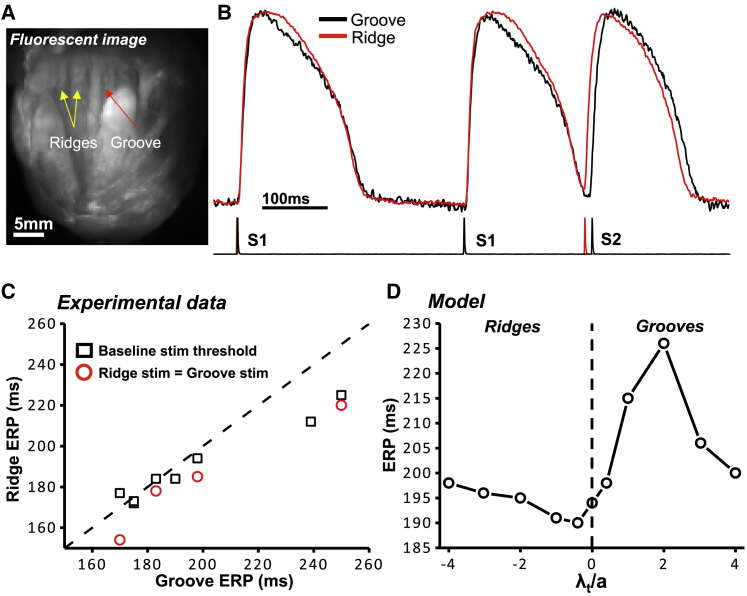
(*A*) A fluorescent image of the rabbit endocardial left-ventricular surface, with arrows showing the locations of ridges and grooves. (*B*) An example of the optically recorded action potentials obtained during the S1-S2 protocol used to determine the ERP; the stimuli waveforms are shown under the action potentials. (*C*) Square black data points: experimental ERPs obtained using the individual minimal stimulus strengths required to elicit capture at each (ridge and groove) location. Circular red data points: experimental ERPs obtained using an equal stimulus strength at each location. The dashed line is of *x* = *y*. (*D*) The computational results for the ERPs in ridge and groove geometries, as parameterized by the nondimensional curvature *λ*_*t*_/*a*. To see this figure in color, go online.

Fig. 4 *A* shows a fluorescent image of one of the rabbit left-ventricular endocardial preparations, showing the locations of the ridges and grooves. Fig. 4 *B* then shows representative experimental action potentials at ridge (*red trace*) and groove (*black trace*) sites during the ERP computation. In the experimental measurements, direct comparison of ERP between sites on the endocardial ridges versus grooves was problematic because of the overall range in ERPs observed. Instead, pairwise comparisons were performed to examine the differences in ERP between groove and ridge locations of the same sample. These are shown in Fig. 4 *C*. As with the case of capture thresholds, the experimental data again suggest that ridge locations are more easily excitable than grooves; in almost all cases, the ERPs recorded in the ridges were lower than in corresponding groove locations, shown by points lying below the dashed *x* = *y* line in Fig. 4 *C*.

It should be noted that two sets of data points (*black squares* and *red circles*) are shown in Fig. 4 *C*. Black squares correspond to the values obtained for the minimal stimulus strengths, as shown in Fig. 3 *C*. Red circles correspond to matched stimulus strengths (equal at both sites). For matched stimulus strengths, all ridge-groove pair recordings showed a lower ERP in the ridge than the groove. For differing stimulations strengths, six out of eight ridge-groove pair recordings showed a lower ERP in the ridge than the groove. Specifically, when stimulated at the minimal strength for capture, a pairwise difference (groove minus ridge) of 7.4 ± 4.2 ms was seen, which increased to 15.8 ± 5.3 ms for matched stimulus strengths.

#### Computational results

As with the case of capture thresholds, the computational model predicted similar trends in ERP as seen in the experimental recordings above. The ERP at the site of the bipolar stimulus was calculated for all the computational geometries. Fig. 4 *D* shows the ERPs versus nondimensional curvature. The value of ERP with zero curvature (or a flat surface, as indicated by the *dashed line*) is ∼195 ms. This increased to 228 ms for a groove with curvature *λ*_*t*_/*a* = 2. Fig. 4 *D* shows that for negative curvature (grooves), although the value of ERP reduces, the magnitude of change is less than in the case of positive curvature, reducing to ∼190 ms. For both large negative and positive curvatures, the values of ERP return toward the zero-curvature case. The reasons for this are similar to the those described above for the minimal stimulus strength. The asymmetrical response of ERP, in contrast to the relatively symmetrical threshold stimulus response (in terms of magnitude of difference around *λ*_*t*_/*a* = 0), is likely due to the highly nonlinear nature of ordinary differential equations (22) governing the excitability of the repolarizing membrane.

### Electrophysiological heterogeneities

#### Computational results

The computational models used in this study were assigned uniform ionic properties throughout, and thus any differences in action potential characteristics could only be driven by electrotonic influences. In both ridge and groove models, the well-known decrease in APD away from the stimulus site was witnessed, following the path of activation (33). However, despite the differences in electrotonic loading near the stimulus site, there were no differences in APD seen at the stimulus site between ridge or groove models, with both giving APDs of approximately 191 ms. Thus, the significant differences in ERP in the computational modeling results were not driven by differences in APD but instead by electrotonic loading effects.

#### Experimental results

**Figure 5:**
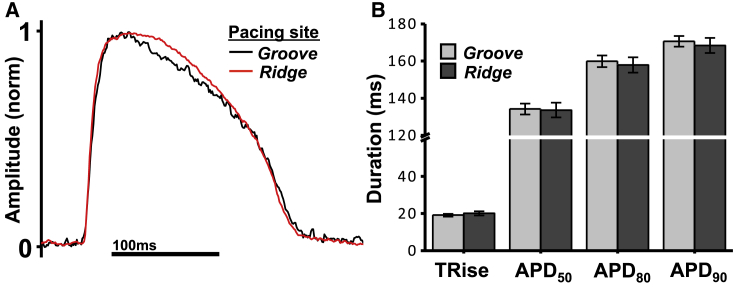
(*A*) Example optical action potential traces recorded from the ridge (*red*) and groove (*black*) sites. (*B*) Quantification of optical action potential characteristics recorded between ridge and groove sites, showing upstroke duration, APD_50_, APD_80_, and APD_90_. To see this figure in color, go online.

Contrary to the computational model, APD was heterogeneous throughout and between the experimental preparations. Fig. 5 *A* compares a representative optical action potential traces from ridge (*red*) and groove (*black*) sites, showing similar morphologies. Fig. 5 *B* then quantifies differences in action potential upstroke duration as well as APD_50_, APD_80_, and APD_90_. No significant differences were seen in action potential characteristics in the experimental preparations between measurements made on ridges and grooves. This suggests that the differences in ERPs are not driven by any intrinsic differences in APD or excitability but instead are due to differences in electrotonic loading around the stimulus site.

### Simulation of strength-interval curves

**Figure 6:**
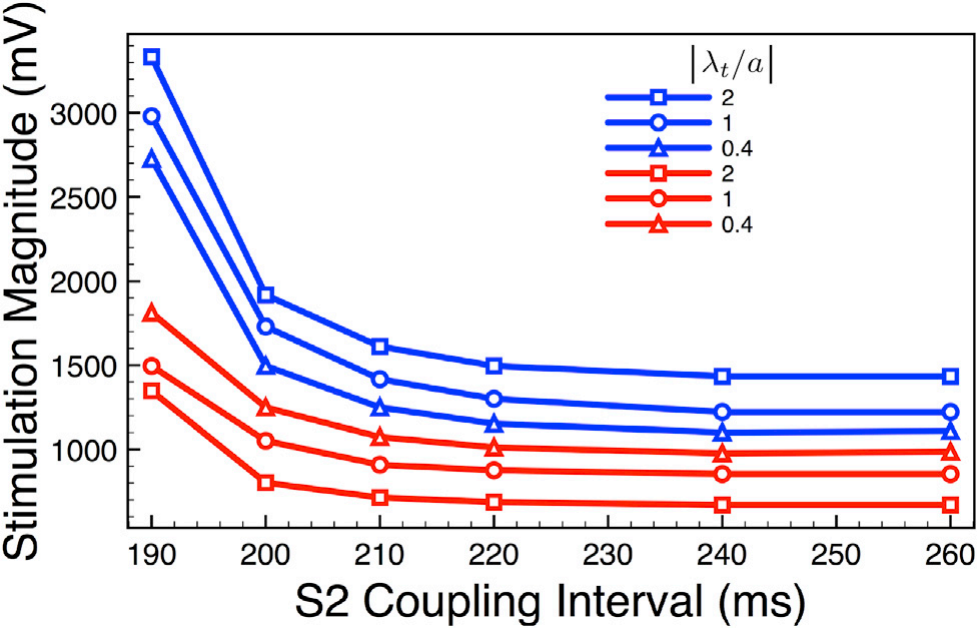
Strength-interval curves for both protrusion (*red*) and groove (*blue*) models, each with three different curvatures with |*λ*_*t*_/*a*| ratios of 2 (*squares*), 1 (*circles*), and 0.4 (*triangles*). To see this figure in color, go online.

The changes found in the sections above in capture threshold and ERP were then further examined within the computational models through the calculation of strength-interval curves, as described in the Methods. Fig. 6 shows strength-interval curves for both protrusion and groove models, each with three different curvatures. Fundamentally, these curves underscore the findings above but with additional key insights. Principally, all groove structures, regardless of curvature, require higher stimulus strengths for tissue capture than protrusions and at all different levels of refractoriness. Furthermore, the ordering of strongest to weakest capture threshold in terms of geometry is also preserved for all S2 timings. Importantly, the differences seen in stimulus capture between protrusions and grooves of similar curvature in Fig. 3 at diastole are significantly augmented when the tissue becomes more refractory. For example, at 260 ms after the initial S1 stimulus (when the tissue is in diastole), there is a difference of less than 0.4 V for a curvature of 1, whereas this increases to almost 1.5 V difference when the tissue is refractory at 190 ms after the S1.

### Simulations of DAD capture

**Figure 7:**
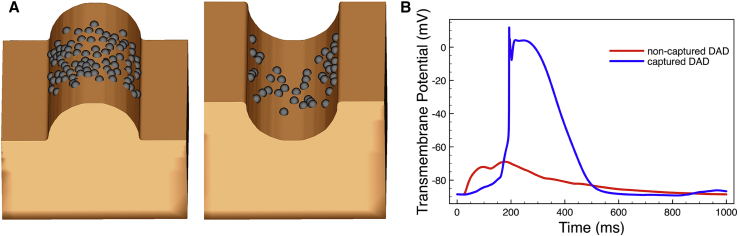
(*A*) DAD-capture events within a protrusion (*left*) and groove (*right*) structure model, both with curvature |*λ*_*t*_/*a*| = 0.4. (*B*) Example traces from single nodes within the tissue protrusion model showing a successful DAD-capture event (*blue*) and an unsuccessful DAD event (*red*) that did not capture. To see this figure in color, go online.

The ability of a stimulus to capture a protrusion structure with a lower capture threshold and/or at increased levels of tissue refractoriness compared to a similar groove structure shown in the analysis above might suggest that the capture of triggered activity is more likely in such a protrusion structure, for which electrotonic loading is lower. Here, we perform simulations of spontaneous DAD capture within a protrusion model, along with a groove model of corresponding radius. Fig. 7 *A* shows that DAD capture occurs preferentially in tissue within the protrusion structure itself (*left*); in the case of a groove, however, fewer capture events occur (*right*). Specifically, for a groove/protrusion structure with a curvature of |*λ*_*t*_/*a* = 0.4|, 11.7% of simulations resulted in DAD capture events occurring within the protrusion model, with just 4.3% of simulations producing successful capture in the groove model. For models with a curvature of |*λ*_*t*_/*a* = 2|, the magnitude of capture events was lower (because there is physically less tissue within the structure capable of producing DADs); however, the relative difference likelihood of DAD capture was higher compared to the larger structure models: 1.4% of simulations produced DAD capture in the protrusion model compared to 0.4% in the groove model. Fig. 7 *B* plots two example traces from the same site on the protrusion model, showing the difference between successful capture (*red*) and unsuccessful capture (*red*) at the same site that a DAD occurred. In the case of unsuccessful capture, the slight elevation of *V*_*m*_ is not sufficient to capture because of a lack of synchrony with surrounding tissue (which is a stochastic process), and the potential returns to baseline.

## Discussion

### Geometrical boundary confinement

Successful capture of a region of tissue depends upon the strength and duration of the stimulating pulse, the state of excitability of the tissue being stimulated, and the local electrotonic loading surrounding the stimulus site. We have shown in earlier works that focal stimulation of passive tissue close to an insulating boundary (such as an internal vessel cavity, endocardium (14), or fibrosis bundle (12)) raises local *V*_*m*_ to higher levels than when the stimulus site is within well-coupled tissue, far from tissue boundaries. Close to such boundaries, electrotonic loading is reduced, and the stimulating current is locally confined around the stimulus site itself; this effect is highlighted in Fig. 1. For a fixed stimulus, electrotonic loading differences cause the change in transmembrane potential to be greater near a ridge than a groove. Therefore, a fixed stimulus may raise the local transmembrane potential above the threshold potential for capture in a ridge earlier than a groove. However, until now, experimental validation of these proposed effects has been lacking.

In the experiments performed in this study, the nonpassive nature of myocardial tissue meant that the stimulus threshold for capture was used as a surrogate for electrotonic loading—for a fixed duration stimulus applied to diastolic tissue, the minimal strength required for capture would be directly related to the degree of local electrotonic loading. The threshold stimulus for capture is dominated by the subthreshold linear response of the membrane, meaning that in pathological scenarios resulting in altered membrane excitability (such as in Brugada syndrome, for example), the curve of the stimulus threshold for capture would likely be translated by some constant. Because of practical restrictions in accessing intramural stimulus locations, sites on the endocardial surface with differences in surface curvature were chosen: endocardial trabeculae ridges (with negative curvature) and grooves (with positive curvature). In the case of the ridge, the tissue just under the stimulus site is more closely surrounded by tissue surface boundaries than in the case of the groove, meaning the electrotonic loading is lower around a ridge than a groove. Our experimental measurements agree qualitatively with the modeling predictions, demonstrating that the minimal threshold for capture is consistently lower in the ridge than the groove. This agrees with our previous modeling predictions regarding boundary confinement in passive models upon focal stimulus (12, 14).

Our findings are also in agreement with a combined experimental and computational study by Bittihn et al. (16), who examined the effects of local anatomical structures on far-field stimulation similar to that used during defibrillation. Here, the authors also demonstrated that *V*_*m*_ would be raised higher close to boundaries of negative curvature because of a reduction in diffusive loading compared to boundaries of positive curvature. However, it is important to note that the findings in (16) were also critically dependent upon the uniformly applied electric field, as discussed in (17).

The computational predictions of the stimulus threshold and ERP versus cylinder radius (or nondimensional curvature) should be considered valid only for the geometries used in this work (a half-cylinder added or subtracted from a flat surface). For instance, if the geometry were a parabolic extrusion or a parabola of revolution, which was not directly attached to (or removed from) a block of myocardial tissue but instead extended out to infinity, we would expect the electrotonic loading effects on ERP and stimulus threshold as |*λ*_*t*_/*a*| → ∞ to be monotonic as in (16) rather than reaching some peak around |*λ*_*t*_/*a*| ≈ 2, as shown in this work. The presence of the underlying myocardial tissue, chosen to replicate the real anatomy of these structures, is thus responsible for the specific trends uncovered here. Furthermore, we nondimensionalized the curvature via the transverse space constant in the graphs in Figs. 3 *D* and 4 *E* to show the generality of the computational results. If the same simulation were repeated on a uniformly scaled geometry, provided the ratios *λ*_*t*_/*a* and *λ*_*t*_/*λ*_*l*_ are fixed, similar trends should be expected. The spacing of the bipolar stimuli may, however, change the gradients of the curves to some degree—this may be more pronounced for the ERP (because of proximal de- and hyperpolarization facilitating break excitations) than for the stimulus threshold in fully excitable tissue.

Note that we did not consider different fiber orientations in the modeling specifically to be consistent with the fiber orientation expected in the experimental preparations (25). If we had performed the simulations with fibers normal to the trabeculation axis, we would get different results for ERP and threshold voltage; however, we would expect similar trends in the results to those shown. To verify the independence of the trends in threshold voltage to the specific fiber-orientation chosen, we performed two-dimensional (2D) isotropic simulations with the bipolar stimuli approximated via surface transmembrane stimuli (results not shown) and indeed observed the same trends with respect to curvature.

### Disparity between the APD and ERP differences

An important physiological functional consequence of the difference in electrotonic loading at different anatomical stimulation sites was the resulting difference in ERP witnessed. This difference in ERP was seen in both modeling and experimental measurements in the absence of any difference in APD, supporting the conclusion that electrotonic loading was driving this difference. Similar findings have been made in recent modeling studies, showing significantly lower ERPs around intramural vessel cavities (14), around external tissue boundaries (14), and near fibrotic bundles (12) compared to well-coupled tissue, again in the absence of any variation in APD. This study therefore provides essential experimental validation of our earlier findings.

As illustrated in Theory of Boundary Confinement, the stimulus current delivered to the endocardial ridge is locally confined to a greater degree than the groove because of the lower diffusive load around the ridge due to the negatively curved boundary. Thus, the “effective” strength of a given stimulus is greater in the case of a ridge than a groove. Therefore, for a given stimulus strength, stimulus on a ridge is able to capture at relatively earlier coupling intervals (as the electrotonic loading is lower, meaning the stimulus can overcome increased states of tissue refractoriness) compared to the groove.

In addition to electrotonic loading, another factor related to excitability that may differ between anatomical stimulus sites is potential heterogeneity in the sodium current (conductance, channel density, etc.). No intrinsic ionic heterogeneity was assigned to the computational model, suggesting that observed variations in ERP are due to excitability as modulated by local electrotonic loading and not due to significant heterogeneity in sodium current or other ion channels. The fact that Fig. 5 also shows that there was no significant difference in action potential upstroke duration between endocardial ridge versus groove sites in experimental recordings, as well as no significant differences in APDs, provides further evidence to support the notion that the capture threshold is entirely driven by differences in loading and not sodium or other ionic heterogeneity.

A potential issue in the experimental recordings is the possibility of stimulating Purkinje cells, which may be more or less likely on a ridge or groove depending upon the specific distribution of the endocardial-bound Purkinje fibers. However, the experimental measurements of ERP were (by definition) attempting to capture at very short coupling intervals. Because of the known longer APD of Purkinje cells compared to ventricular myocytes, Purkinje tissue would still be refractory at the coupling intervals at which capture was possible in ventricular myocytes. Thus, heterogeneity in tissue type should not be contributing to the differences in ridge and groove recording locations seen here.

### Implications for arrhythmogenesis

An important consequence of regions of reduced electrotonic loading and subsequent increased excitability is their potential increased vulnerability to successful capture of focal ectopic beats due to afterdepolarizations or abnormal automaticity, for example. Indeed, we showed in computational simulations that in the setting of tissue that is susceptible to SCR, DAD capture is more likely on sites on the endocardium within ridges as opposed to grooves. In a ridge, less depolarizing current is required to capture (lower capture threshold), and there exists a larger window of vulnerability (due to reduced ERP), making capture by such weakly depolarizing events more likely. This was shown in Fig. 7 as an increase in the percentage of simulations showing spontaneous capture in protrusion structures compared to corresponding groove structures. Uncovering such a mechanism of increased likelihood of ectopic capture in relation to local tissue structure provides important mechanistic insight regarding the largely unresolved issue of successful capture of isolated DAD events. Up until now, the primary suggested location for these sites has been within thin, cable-like Purkinje fibers (6, 31). Here, we show that endocardial protrusions have electrotonic loading properties that, because of their relatively small radii, indeed approach such 2D-like structures, making them much more susceptible to DAD capture compared to groove structures. We note, however, that investigating DAD capture in computational models and experiments is problematic because it is a stochastic event. The likelihood of capture can be increased by artificially synchronizing Ca overload, which helps to overcome the local electrotonic source-sink mismatch. Here, however, we facilitated DAD-capture through a reduction in the overall tissue conductivity (reducing overall loading) to an extent at which DAD capture occurred in our 3D models. This allowed a direct comparison between the likelihood of DAD capture between our anatomical models with different electrotonic loading properties. The changes in conduction in our models were made to approximate the downregulation and phosphorylation of connexin43, as well as increased myocardial fibrosis, that may occur in severe heart failure conditions in which DAD capture may be most likely. Our goal was not to precisely model heart failure conditions but rather to assess how different anatomical structures such as endocardial ridges and grooves can alter electronic load and favor or prevent DAD capture in a scenario of severely reduced conduction. The effects of similar severe conduction reductions (up to 200-fold compared to well-coupled myocardium) have been investigated previously in computational modeling studies aiming to investigate the effects of severely impaired conduction on impulse propagation in the context of heart failure (34, 35), which were shown to be similar to measurements from experimental rat preparations (36). Understanding more about capture of such triggered events is important in the mechanisms underlying the formation of premature ventricular complexes, which may be arrhythmogeneic. This insight may provide important guidance to clinicians during ablation procedures of arrhythmias that are known to be of a focal nature, particularly as catheter laboratory navigation systems become increasingly more advanced.

### Implications for external electrical stimulation

The finding that endocardial trabeculae ridges require lower stimulus thresholds for capture than grooves provides insight as to how minimal stimulus currents may be modulated based upon specific placement of an external pacing electrode on the tissue surface. Such findings are important in preclinical experiments, in which exact knowledge of stimulation currents and their heterogeneity based on anatomical locations may be vitally important for delivering specific pacing protocols and making intricate electrophysiology measurements. Furthermore, in clinical electrophysiology procedures, specific pacing strategies are delivered to the endocardial tissue surface via catheterized electrodes in addition to measurements of refractoriness. Increasingly, such electrophysiological data is combined with anatomical information from clinical scans, and in the future, knowledge of how differences in catheter location may affect the measurements made could have implications for interpreting the underlying electrophysiological substrate.

A wealth of simulation and experiment literature exists investigating the bidomain effects of unipolar and bipolar stimulus with respect to the excitability mechanisms of tissue capture, principally mediated via virtual-electrode-induced polarizations and the role of make-and-break excitations (37). In Fig. 3 *D*, we showed how the regions of tissue that experience depolarizing and hyperpolarizing effects of the bipolar electrode configuration are more extensive in the ridge case compared to the groove case because of the increased boundary confinement of the stimulation current along the trabecular ridge. The increased magnitude of these regions of opposite tissue polarization in the ridges thus explains the increased excitability during bipolar stimulus application conducted in this study. Although cathodal make excitation was the main mechanism of capture seen in these simulations, break excitation effects—in which the regions of depolarization under the anode initially conduct through the newly made regions of hyperpolarization under the anode upon stimulus cessation—would be more prominent when the stimulus is applied at increasing stages of refractoriness (at higher strengths than applied here). The interaction between regions of de- and hyperpolarization induced by the bipolar electrode has important synergies with other studies examining direct unipolar stimulation (37) by an insulated plunge electrode (38) or incorporating the effects of local tissue injury (39), as well as by application of a 9-V battery (40), in addition to far-field virtual electrodes induced around fine-scale anatomical structures such as intramural clefts (41) and blood vessels (17, 27, 42, 43).

It should be noted that the findings from this work do not have immediate clinical relevance in relation to pacing from implanted cardiac rhythm management devices. In such devices, pacing electrodes are often screwed deep into the myocardium via active fixation to secure them, causing local injury (39) and becoming locally encapsulated by fibrosis, which generates a different physiological environment to that represented by our models or in the experiments performed in this study. However, similar isolated surface stimulation procedures, such as those represented in this work, could be of key relevance to the rapidly growing field of cardiac optogenetics (5). If such advances continue, it may be possible to selectively transfect regions of myocardium with channelrhodopsin that under correct illumination may function as internal pacemakers or alternatively as pacing sites to terminate arrhythmias. In this scenario, illumination would most likely be directed on specific regions of the endocardium tissue surface, whereas transfected cells may well be distributed directly within the myocardium itself. Consequently, detailed knowledge of how capture thresholds and timings may vary in response to local anatomical environment and corresponding electrotonic loading may be useful for optimizing the therapies based on this technology.

### Limitations

The modeling considers only one, homogeneous fiber orientation—parallel to the cylinder axis—because this is known to be representative of the real fiber directions (25); other fiber orientations are predicted to lead to changes in magnitudes of the effects observed but are unlikely to change the overall trends. As a test, we conducted equivalent 2D isotropic monodomain simulations with the bipolar stimuli approximated via surface transmembrane stimuli (results not shown) and observed similar trends in the stimulus threshold as a function of cylinder curvature. In the 3D simulations in this work, we did not resolve the electrodes themselves or consider their effect on the external bath (and thus the electric field in this region) and its interaction with the tissue. This was done for two reasons: 1) the virtual electrode magnitudes are largest where the electrodes are in contact with the tissue surface (as we have done in the simulations), and thus we expect the electrotonic loading in these regions to dominate the excitatory effects; and 2) only the tips of the (physical) electrodes used in the experiment were not insulated, and thus the computational representation of the electrodes was reasonable. During the experimental measurements, it is possible that there could have been very small differences in electrode contact for the different geometries considered. However, given the very lightweight nature of the wire electrodes used relative to the anatomical structures along with the care taken to ensure that there was no mechanical distortion of the tissue surface, we believe any systematic bias in the conditions regarding electrode contact was minimized. Finally, as mentioned above, considering idealized bipolar stimulus does not account for the specific active-fixation helix used in the majority of implanted cardiac device leads, nor does it account for large clinical pacing electrodes that may not fit within the small structures investigated here and may cause local tissue deformation, affecting the geometry investigated. However, our idealized bipolar arrangement is a faithful representation of bipolar catheter pacing during exploratory electrophysiology procedures and pacing in experimental preparations, in addition to conveying important fundamental information regarding the biophysics of stimulus application to cardiac tissue. We also note that in practical clinical scenarios, the orientation of the catheter or lead with respect to the surface will vary depending on anatomical position and may not generate a symmetrical dipole as represented in this work. Orientating the lead at other angles including orthogonal to the surface will alter the magnitude of the polarization of the tissue surface and the effective stimulus for a given applied potential difference; however, this will affect the tissue response in both ridges and grooves in a similar manner.

## Author Contributions

Designed study, A.C., M.J.B., F.O.C., G.S., A.K., and R.M.; performed simulations, A.C., F.O.C., and M.J.B.; performed experiments, A.K. and R.M.; analyzed data, A.C., M.J.B., F.O.C., and A.K.; drafted manuscript, A.C., M.J.B., F.O.C., A.K., R.M., and G.S.
